# Amelogenin Downregulates Interferon Gamma-Induced Major Histocompatibility Complex Class II Expression Through Suppression of Euchromatin Formation in the Class II Transactivator Promoter IV Region in Macrophages

**DOI:** 10.3389/fimmu.2020.00709

**Published:** 2020-04-21

**Authors:** Karen Yotsumoto, Terukazu Sanui, Urara Tanaka, Hiroaki Yamato, Rehab Alshargabi, Takanori Shinjo, Yuki Nakao, Yukari Watanabe, Chikako Hayashi, Takaharu Taketomi, Takao Fukuda, Fusanori Nishimura

**Affiliations:** ^1^Division of Oral Rehabilitation, Department of Periodontology, Faculty of Dental Science, Kyushu University, Fukuoka, Japan; ^2^Dental and Oral Medical Center, Kurume University School of Medicine, Kurume, Fukuoka, Japan

**Keywords:** amelogenin, periodontal tissue regeneration, macrophages, major histocompatibility complex class II, interferon gamma, class II transactivator, immunosuppression

## Abstract

Enamel matrix derivatives (EMDs)-based periodontal tissue regenerative therapy is known to promote healing with minimal inflammatory response after periodontal surgery, i. e., it promotes wound healing with reduced pain and swelling. It has also been reported that macrophages stimulated with amelogenin, a major component of EMD, produce various anti-inflammatory cytokines and growth factors. We previously found that stimulation of monocytes with murine recombinant M180 (rM180) amelogenin suppresses major histocompatibility complex class II (MHC II) gene expression using microarray analysis. However, the detailed molecular mechanisms for this process remain unclear. In the present study, we demonstrated that rM180 amelogenin selectively downmodulates the interferon gamma (IFNγ)-induced cell surface expression of MHC II molecules in macrophages and this mechanism mediated by rM180 appeared to be widely conserved across species. Furthermore, rM180 accumulated in the nucleus of macrophages at 15 min after stimulation and inhibited the protein expression of class II transactivator (CIITA) which controls the transcription of MHC II by IFNγ. In addition, reduced MHC II expression on macrophages pretreated with rM180 impaired the expression of T cell activation markers CD25 and CD69, T cell proliferation ability, and IL-2 production by allogenic CD4^+^ T lymphocytes in mixed lymphocyte reaction assay. The chromatin immunoprecipitation assay showed that IFNγ stimulation increased the acetylation of histone H3 lysine 27, which is important for conversion to euchromatin, as well as the trimethylation of histone H3 lysine 4 levels in the CIITA promoter IV (p-IV) region, but both were suppressed in the group stimulated with IFNγ after rM180 treatment. In conclusion, the present study shows that amelogenin suppresses MHC II expression by altering chromatin structure and inhibiting CIITA p-IV transcription activity, and attenuates subsequent T cell activation. Clinically observed acceleration of wound healing after periodontal surgery by amelogenin may be partially mediated by the mechanism elucidated in this study. In addition, the use of recombinant amelogenin is safe because it is biologically derived protein. Therefore, amelogenin may also be used in future as an immunosuppressant with minimal side effects for organ transplantation or MHC II-linked autoimmune diseases such as type I diabetes, multiple sclerosis, and rheumatoid arthritis, among others.

## Introduction

Periodontitis is the leading cause of tooth loss in adults and is characterized by chronic inflammation of the periodontal tissue due to pathogenic periodontal bacterial infection. Periodontal lesions progress with slow tissue destruction, eventually leading to tooth loss (1, 2). Traditional periodontal interventions have only sought to prevent the progression of periodontal disease; however, there has been a strong demand in recent years for therapeutic strategies that aim to rebuild healthy periodontal tissue. Many periodontal tissue regenerative methods have thus been developed to meet this demand (3). One such treatment strategy is the use of enamel matrix derivatives (EMDs) such as Emdogain®, which are now widely used in periodontal surgery. This strategy was developed based on the concept of mimicking the specific developmental environment for teeth, and has achieved positive clinical results (4–6). Emdogain® is an EMD that is extracted and purified from porcine tooth germs at the age of 6 months. Amelogenin is the most abundant protein present in Emdogain® and is central to EMD activity, apart from enamelin, ameloblastin, tuftelin, differentiation-inducing factors, and growth factors (7).

Amelogenin is an extracellular matrix (ECM) protein of size 20–25 kDa that is secreted primarily by ameloblasts during tooth development. Amelogenin contains a hydrophobic region at the N-terminus and a hydrophilic region at the C-terminus, but overall, the protein's biochemical properties are dominated by the proline-rich hydrophobic region. Amelogenin is heavily secreted during enamel formation and is composed of a molecular aggregate called an amelogenin nanosphere. Hydrophobic amelogenin nanospheres in the ECM act as a scaffold for ameloblasts during the development of tooth germs, and are degraded by serine proteases and enamelysin (matrix metalloproteinase-20) upon calcification of the enamel (8, 9). Fragments of amelogenin nanospheres are also known to promote the growth of crystalline hydroxyapatite. Furthermore, amelogenin is thought to be involved in the formation of cementum through deposition on the dentin of the root. In fact, enamel and cementum hypoplasia has been observed in the oral cavity of amelogenin-deficient mice (10). In recent years, a number of studies have claimed that ECMs including amelogenin control cellular functions such as proliferation, survival, migration, and differentiation, and that they play an important role in regulating tissue remodeling. They are involved in homeostatic tissue anabolic and catabolic processes, rather than maintaining a stable structure during biological processes such as development and wound healing (11).

EMD-based periodontal tissue regenerative therapy is known to promote healing with minimal inflammatory response after periodontal surgery, i.e., it promotes wound healing with markedly reduced pain and swelling (12). It has also been reported that macrophages stimulated with amelogenin produce various anti-inflammatory cytokines and growth factors such as interleukin-10 (IL-10), vascular endothelial growth factor (VEGF), and insulin-like growth factor-1 (IGF-1) (13). However, most studies discussing the relationship between amelogenin and wound healing have been dominated by phenomenology, and few have provided a detailed molecular mechanism of amelogenin's role in wound healing.

In generally, periodontal inflammation involves an immune response to periodontal pathogenic bacteria initiated by the recognition and presentation of foreign antigens via macrophages, dendritic cells, or B lymphocytes. Foreign antigens induce adoptive immune responses and activate a pro-inflammatory environment that shifts the differentiation of CD4^+^ T lymphocytes toward a Th1 phenotype. These Th1 cells produce the pro-inflammatory cytokines interferon gamma (IFNγ) which promotes bactericidal activity, secretion of other pro-inflammatory cytokines, and tumor suppression by macrophages. In addition, IFNγ enhances the expression of many crucial molecules on the surface of antigen-presenting cells, such as major histocompatibility complex class I (MHC I), MHC II, and costimulatory molecules CD80/86 at the transcriptional level (14–19). In particular, the transcription of MHC II by IFNγ is controlled by transcriptional master regulator, class II transactivator (CIITA) (20–22). CIITA assembles a complex of transcription factors such as regulatory factor X (RFX), cyclic-AMP-responsive-element-binding protein (CREB), and nuclear transcription factor Y (NFY) at the MHC II gene promoters (23).

Some immunosuppressant drugs exert its immune tolerance by inhibiting the expression of MHC II molecules (24–26), and the reduced MHC II expression induces wound healing through the transformation of macrophages (27–30). We previously found that the stimulation of monocytes with murine recombinant M180 (rM180) amelogenin, a major component of EMD, suppresses MHC II gene expression using microarray analysis (31). However, the detailed molecular mechanisms for this process remain unclear. The elucidation of this mechanism contributes to gaining better insight into the resolution of inflammation by EMD during periodontal tissue regenerative processes, and also in understanding the potential role of amelogenin in the regulation of immunological rejection for future establishment of novel tolerogenic immunosuppressive therapy for organ transplantation. Hence, the aims of this study were to examine in detail the molecular mechanisms underlying amelogenin action on macrophages and to verify its effects on the antigen presentation of IFNγ-induced MHC II.

## Materials and Methods

### Cell Cultures

The THP-1 human monocytic cell line and the RAW264.7 murine macrophage cell line were purchased from RIKEN BioResouce Center (Ibaraki, Japan). THP-1 cells were maintained in RPMI-1640 medium (Nacalai Tesque, Kyoto, Japan) containing 10% heat-inactivated fetal bovine serum (FBS), penicillin, and streptomycin at 37°C in a 5% CO_2_ incubator. Cells were sub-cultured every 48–72 h, inoculum being 5 × 10^5^/mL, and cell viability (>95%) was confirmed by trypan blue exclusion. After the THP-1 cells were stimulated with 50 nM phorbol-myristate-acetate (PMA) (Sigma Aldrich, St. Louis, MO, USA) for 24 h, they were washed with phosphate-buffered saline (PBS, pH 7.4) and replaced with PMA-free RPMI-1640 medium with FBS for another 24 h. These differentiated macrophages were pretreated with or without 10 μg/mL recombinant murine M180 amelogenin (rM180) for 24 h, and then stimulated with 2.5 ng/mL recombinant human IFNγ (PeproTech, Rocky Hill, NJ, USA) for 24, 36, and 48 h. The murine macrophage cell line RAW264.7 (American Type Culture Collection (ATCC), Manassas, VA, USA) was maintained in Dulbecco's modified Eagle's medium (DMEM) (Nacalai Tesque) containing 10% heat-inactivated FBS, penicillin, and streptomycin. RAW264.7 cells were stimulated in same manner as described for THP-1 cells using 2.5 ng/mL recombinant mouse IFNγ (BioLegend, San Diego, CA, USA) and 10 μg/mL rM180.

### Animals

The female C57BL/6 mice (8 week-old) and BALB/c mice (8 week-old) were purchased from Charles River Laboratories Japan, Inc. (Yokohama, Japan). Experiments were approved by Animal Care and Use Committee of Kyushu University (Permit Number: A19-285-0).

### Preparation of Recombinant Murine M180 Amelogenin

The cloning and expression of a glutathione *S*-transferase (GST) full-length M180 amelogenin fusion construct, and the purification of rM180 were previously described (32). Briefly, full-length cDNA for mouse amelogenin (M180) was cloned into the vector and transformed into competent *Escherichia coli*. The bacterial pellets containing recombinant GST-rM180 were purified, and on-column cleavage of rM180 from the GST portion of the fusion protein was carried out with the PreScission protease (GE Healthcare, Boston, MA, USA). Endotoxin removal from rM180 was confirmed by the Limulus amebocyte lysate assay (endotoxin level: 10 μg of rM180 < 0.03 EU).

### Flow Cytometry

For flow cytometry analysis, THP-1 cells, RAW264.7 cells, and mouse bone marrow macrophages were seeded in a 6-well plate at a density of 1 × 10^6^ cells/well. After stimulation with IFNγ, or IFNγ after rM180 treatment for 24 h, cells were detached with Accutase (Nacalai Tesque), centrifuged at 300 *g* for 5 min, and washed with stain buffer (BD Pharmingen, San Diego, CA, USA). Cells were blocked with human TrusStain FcX (BioLegend) for 10 min at room temperature. To determine the surface expression of target molecules, cells were then washed and stained with PE anti-human HLA-DR (BioLegend), PE anti-human CD86 (BioLegend), Alexa Fluor 488 anti-human HLA-A, B, C (BioLegend), and FITC anti-mouse I-A^d^ (BioLegend). Isotype controls were used to confirm antibody specificity. Cells were incubated in the dark for 30 min at 4°C and analyzed using a BD FACSVerse flow cytometer (BD Biosciences, San Diego, CA, USA). Data were processed using FlowJo^TM^ (v10.5.3) software (BD Biosciences).

### Confocal Microscopy Experiments

PMA-differentiated THP-1 cells were seeded onto a glass slide, and stimulated with rM180 for 0, 2, 5, 15, 30 min, 1, 12, and 24 h to observe the intracellular uptake of rM180. After stimulation, cells were fixed using 4% paraformaldehyde (PFA) for 20 min, blocked with Blocking One Histo (Nacalai Tesque) for 5 min at room temperature, treated with 0.5% Triton X-100 (Junsei, Tokyo, Japan), which penetrated into the cells, for 10 min at room temperature, and stained with primary anti-amelogenin (F-11): sc-36528 (1:250; Santa Cruz Biotechnology, Inc, Dallas, TX, USA) antibodies overnight at 4°C. To observe the cell surface expression of MHC II molecules, cells were stained with primary anti-HLA-DR (L243): sc-18875 (1:250; Santa Cruz Biotechnology, Inc.). Subsequently, Alexa Fluor® 594 goat anti-mouse IgG (minimal x-reactivity) (1:1000; BioLegend) was used as secondary antibody for 2 h in the dark at room temperature. The nucleus was stained using SlowFade® Diamond Antifade Mountant with DAPI (Life Technologies, Waltham, MA, USA). The images were analyzed by ZEISS LSM700 (Carl Zeiss, Oberkochen, Germany) and ZEN 2012 software.

### RNA Isolation, cDNA Synthesis, and Quantitative PCR

Total RNA was isolated from stimulated or unstimulated macrophages using ISOGEN II (Nippon Gene, Tokyo, Japan). The RNA was quantified by NanoDrop (Thermo Fisher Scientific, Waltham, MA, USA). PrimeScript RT Master Mix (Takara Bio, Otsu, Japan) was used to generate first-strand cDNA. The gene expression level was quantified using Applied Biosystems StepOnePlus^TM^ Real-Time PCR System (Life Technologies, Waltham, MA, USA) according to the attached protocol using KAPA SYBR® FAST qPCR Kit (Nippon Genetics, Tokyo, Japan). PCR was performed as per following program: 95°C for 3 min, 40 cycles of 95°C for 3 s, and 60°C for 30 s. Glyceraldehyde-3-phosphate dehydrogenase (GAPDH) was used as an internal control. The relative expression levels of HLA-DR and CIITA were calculated using the ΔΔCt method. The PCR primer sequences were as follows; HLA-DR, 5′-GGACAAAGCCAACCTGGAAA-3′ (forward) and 5'-AGGACGTTGGGCTCTCTCAG-3′ (reverse); CIITA, 5′-CCGACACAGACACCATCAAC-3′ (forward) and 5′-CTTTTCTGCCCAACTTCTGC-3′ (reverse); IFNGR1, 5′-TCCTCAGTGCCTACACCAACTAATG-3′ (forward) and 5'-CTGGATCTCACTTCCGTTCATTCTC-3′ (reverse); and GAPDH, 5′-CTTTTCTGCCCAACTTCTGC-3′ (forward) and 5′-GTCATACCAGGAAATGAGC-3′ (reverse).

### Western Blots

1 × 10^6^ cells of THP-1 (3 × 10^6^ cells were used to detect CIITA) stimulated with rM180 and IFNγ were lysed and cell extracts representing the total protein content of the cell were separated by 10% sodium dodecyl sulfate-polyacrylamide gel, and transferred to a polyvinylidene difluoride membrane (40 min, 16 V). Following this, the membranes were blocked with 5% skimmed milk in Tris-buffered saline Tween (TBS-T, 20 mM Tris-HCl, pH 7.4, 137 mM NaCl, and 0.1% Tween 20) for 30 min. Subsequently, the membranes were incubated overnight at 4°C with specific antibodies: anti-CIITA (7-1H): sc-13556 (1:1000, Santa Cruz Biotechnology), anti-IRF-1: 11335-1-AP (1:1000, Proteintech, Chicago, IL, USA), anti-STAT1 (1:1000, Cell Signaling Technology, Danvers, MA, USA), anti-phospho-STAT1 (1:1000, Santa Cruz Biotechnology), anti-JAK2 (D2E12) (1:1000, Cell Signaling Technology), anti-phospho-JAK2 (Tyr1007/1008) (C80C3) (1:1000, Cell Signaling Technology), and anti-β-actin (1:1000, Cell Signaling Technology). The membrane was washed five times with TBS-T buffer and exposed to horseradish peroxidase-conjugated secondary antibody. After washing with TBS-T, the membrane was chemiluminescently detected using Chemi-Lumi One Super (Nacalai Tesque) and developed by ImageQuant LAS 4000 (GE Healthcare, Boston, MA, USA). Densitometric analysis of bands was performed using the Image J program (NIH, Bethesda, MD, USA).

### Chromatin Immunoprecipitation (ChIP) Assay

THP-1 (8 × 10^6^) cells were seeded in a 15-cm dish. To cross-linked chromatin, cells were fixed in 4% PFA for 10 min. The ChIP assay was performed using the SimpleChIP® Enzymatic Chromatin IP Kit (Agarose Beads) (Cell Signaling Technology) according to the manufacturer's recommendations. To analyze histone modification, the following antibodies were used: anti- Histon H3 (D2B12) XP® (ChIP formulated) #4620 for positive control, anti- normal rabbit IgG #2729 for negative control, and anti-tri-methyl-histone H3 (Lys9) (D4W1U) (Cell Signaling Technology), anti-AbFlex® Histon H3K4me3 (Active Motif, Carlsbad, CA, USA), anti-AbFlex® Histon H3K27ac (Active Motif), anti-AbFlex® Histon H3K9me3 (Active Motif), anti-AbFlex® Histon H3K27me3 (Active Motif). Purified DNA was analyzed by quantitative real-time PCR using specific primers for CIITA promoter IV (p-IV), 5′-CAGTTGGGATGCCACTTCTGA-3′ (forward) and 5'- TGGAGCAACCAAGCACCTACT-3′ (reverse); and GAPDH, 5′-TACTAGCGGTTTTACGGGCG-3′ (forward) and 5′-TCGAACAGGAGGAGCAGAGAGCGA-3′. Primers for GAPDH promoter were used as negative control. PCR reactions include the positive control histone H3 sample, the negative control normal rabbit IgG sample, a serial dilution of the 2% input chromatin DNA (undiluted, 1:5, 1:25, 1:125) to create a standard curve and determine the efficiency of amplification. Quantitative PCR results were analyzed using the software provided with the real-time PCR machine. The IP efficiency were manually calculated using the Percent Input Method and the equation shown below. With this method, signals obtained from each immunoprecipitation are expressed as a percent of the total input chromatin.

$$\begin{array}{l} \text{Percent Input}=2\text{\% }\times \;2^{\left(\text{C}\left[\text{T}\right]2\text{\% Input Sample}-\text{C}\left[\text{T}\right]\text{ IP Sample}\right)}\\ \text{ C}\left[\text{T}\right]={\text{C}}_{\text{T}}=\text{Threshold cycle of PCR reaction} \end{array}$$

### Mixed Lymphocyte Reaction (MLR)

Mouse bone marrow-derived macrophages were generated from bone marrow mononuclear cells from wild-type C57BL/6 mice. Briefly, femurs and tibiae were collected from 8 week-old mice. After removing bone-adjacent muscles, marrow cells were extracted by flushing RPMI-1640 medium through the bone interior. The cells were then suspended on RPMI-1640 medium containing 10% heat-inactivated FBS, and 40 ng/ml mouse M-CSF (Miltenyi Biote, Bergisch Gladbach, Germany) and plated on 10 cm-dishes. On days 3 and 5, the cells were refed. On day 8, cells were harvested and the expression of monocyte (F4/80^+^, CD11b^+^) markers was analyzed by flow cytometry using APC anti-mouse F4/80 (BioLegend) and PE anti-mouse CD11b (BioLegend). The cells were seeded in 6-well (1 × 10^6^ cells/well) and 96-well plates (5 × 10^5^ cells/well). Cells were stimulated either with IFNγ, or IFNγ after rM180 treatment. After cells were washed with PBS and stimulated with IFNγ for another 24 h, CD4^+^ T cells were added. CD4^+^ T cells were isolated from the spleen of BALB/c mice by positive selection using MojoSort^TM^ Mouse CD4 T Cell Isolation Kit (BioLegend). CD4^+^ T cells were co-cultured with mouse bone marrow-derived macrophages in a 1:1 ratio. Additionally, we cultured only CD4^+^ T cells as a control. After 48 h of co-culture in 6-well plates, T cell activation was analyzed by flow cytometry using APC anti-mouse CD25 (BioLegend), FITC anti-mouse CD69 (BioLegend), and PE anti-mouse CD4 (BioLegend). For carboxyfluorescein succinimidyl ester (CFSE)-based T cell proliferation assay, isolated CD4^+^ T cells were labeled with CFSE using CFSE Cell Division Tracker Kit (BioLegend). T cells (1 × 10^6^ cells) were co-cultured with same number of bone marrow-derived macrophages stimulated with IFNγ after rM180 treatment. After 48 h, cells were stained with PE anti-mouse CD4, and then CFSE dilution was measured by flow cytometry. The IL-2 concentration in the supernatant samples was measured using Legend Max^TM^ Mouse IL-2 ELISA Kit (BioLegend) after collecting the 72 h-culture supernatant samples.

### Statistical Analysis

The data were quantified, and the average value and its standard error (SD) were calculated. All values were expressed as mean ± SD. Comparison between the groups was evaluated using One-way ANOVA or student's *t*-test. *P*-values <0.05 were considered statistically significant.

## Results

### rM180 Amelogenin Downregulated the Expression Profiles of IFNγ-Induced MHC II but Not MHC I or CD86 on Human THP-1 Cells and Murine RAW264.7 Cells

**Figure 1:**
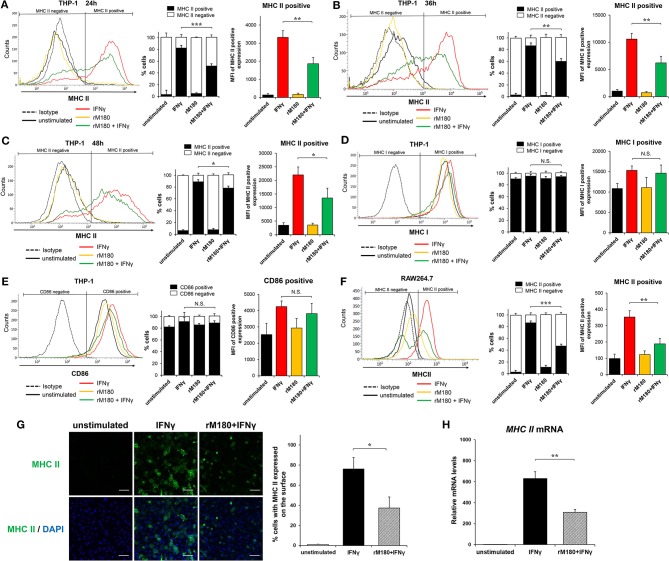
rM180 amelogenin inhibits IFNγ-induced expression of MHC II molecules on human THP-1 macrophages and mouse RAW264.7 cells. **(A–E)** Human THP-1 cells were incubated with rM180 (10 μg/mL) for 24 h, washed, and stimulated with IFNγ (2.5 ng/mL) for 24 h **(A,D,E)**, 36 h **(B)**, and 48 h **(C)**. MHC II **(A–C)**, MHC I **(D)**, and CD86 **(E)** surface expression (left) was evaluated by flow cytometry. Representative histograms are shown in left panel. **(A–C)** Quantification of cells expressing MHC II (MHC II positive cells) or not (MHC II negative cells) is shown in median panel. Quantification of histograms is shown in right panel. Bar graphs represent the mean fluorescence intensity (MFI) of MHC II positive expression. **(F)** Murine RAW264.7 cells were incubated with rM180 for 24 h, washed, and stimulated with IFNγ for 24 h. MHC II surface expression was evaluated by flow cytometry. **(G)** The cells were fixed and stained with the anti-human MHC II antibody, followed by an Alexa 488 secondary antibody (green). Nuclei were stained with DAPI dye (blue). All confocal images are representatives of experiments conducted in triplicates. Quantification of MHC II surface expression (right). Data are expressed as percentage of cells with MHC II on the cell surface. The number of cells per experimental group was 300. Scale bars: 100 μm. **(H)** Total RNA was isolated and analyzed for MHC II mRNA expression by quantitative real-time PCR. Data are represented as fold increase in expression relative to GAPDH. N.S., not significant. The significance of differences between groups was determined by one-way ANOVA/Tukey's test: ^*^P < 0.05; ^**^P < 0.01; ^***^P < 0.001 vs. IFNγ. Data represent mean ± SD. Similar results were obtained in six independent experiments.

We previously demonstrated that rM180 amelogenin suppresses IFNγ-induced MHC II gene expression in monocytic cell lines using microarray analysis (31). To clarify whether rM180 regulates IFNγ-induced MHC II expression in macrophages, THP-1 cells pretreated with or without rM180 for 24 h were stimulated with IFNγ, and then flow cytometry was used to analyze the cell surface expression of MHC II molecules. Furthermore, we distinguished cells expressing MHC II (MHC II positive cells) or not (MHC II negative cells) (Figures 1A–C). THP-1 cells strongly expressed cell surface MHC II upon stimulation with IFNγ for 24, 36, and 48 h (Figures 1A–C). However, cells stimulated with IFNγ after rM180 treatment showed an ~50% reduction in the cell surface positive expression of MHC II compared with the IFNγ-only stimulation control at all time-points (right panel) although rM180^+^ IFNγ gradually decreased the negative expressing population in a time-dependent manner (median panel, Figures 1A–C). In contrast, cell surface expression levels of MHC I and CD86 did not differ between both groups (Figures 1D,E). The selective suppression of MHC II cell surface expression by rM180 was also validated in the mouse macrophage strain RAW264.7 cells (Figure 1F). Cell surface MHC II expression in macrophages was also visualized by immunofluorescence and confocal laser microscopy (Figure 1G). MHC II expression was seen in 80% of cells stimulated with IFNγ only while it was almost absent in unstimulated cells, and showed ~50% reduction in cells stimulated with rM180 + IFNγ (Figure 1G). Quantitative real-time PCR also demonstrated that cells stimulated with rM180 + IFNγ had a similar reduction in MHC II gene expression relative to cells stimulated only with IFNγ (Figure 1H). Overall, these results demonstrated that rM180 amelogenin selectively downmodulates the IFNγ-induced cell surface expression of MHC II molecules and this mechanism mediated by rM180 appeared to be widely conserved across species.

### rM180 Accumulated in the Nucleus of THP-1 Macrophages

**Figure 2:**
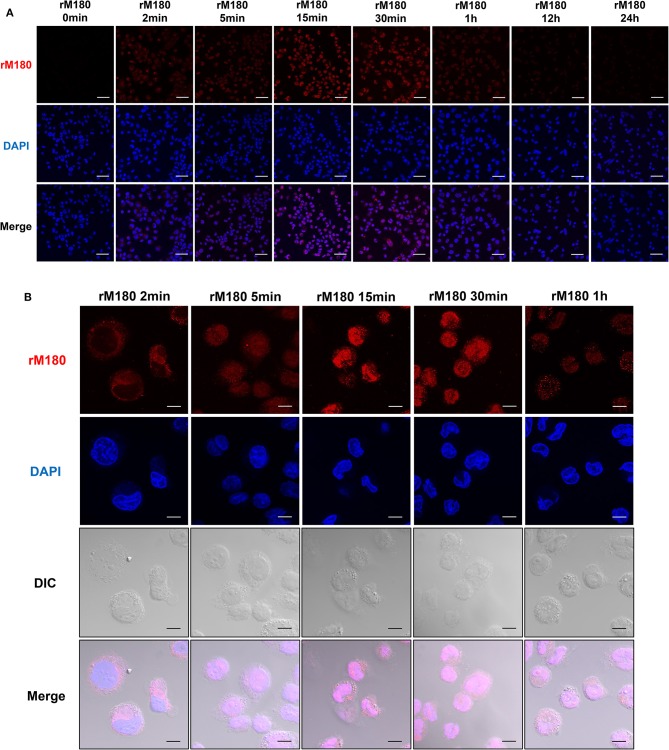
Cellular uptake of rM180 amelogenin in macrophages. **(A)** Time course confocal images of rM180 amelogenin internalization. PMA-differentiated THP-1 cells were incubated with rM180 for 0, 2, 5, 15, 30 min, 1, 12, or 24 h, respectively. The cells were stained with an anti-amelogenin antibody followed by an Alexa 594 secondary antibody (red). Nuclei were stained with DAPI dye (blue). All confocal images are representatives of experiments conducted in triplicate. Scale bars: 50 μm. **(B)** PMA-differentiated THP-1 cells were incubated with rM180 for 2, 5, 15, 30 min, and 1 h, respectively. The cells were stained with an anti-amelogenin antibody (red). Nuclei were stained with DAPI dye (blue). DIC, differential interference contrast. All confocal images are representatives of experiments conducted in triplicate. Scale bars: 10 μm.

Next, we observed the cellular uptake of rM180 amelogenin using confocal laser microscopy. rM180 showed cytoplasmic localization in macrophages at 2 min post-treatment, and subsequent uptake was initiated into the nucleus at 5 min (Figures 2A,B). rM180 accumulated in the nucleus at 15 min after stimulation, with the highest fluorescence intensity observed at 15–30 min (Figures 2A,B). rM180 fluorescence was no longer observable after 12–24 h (Figure 2A).

### Decreased MHC II Expression on Murine Bone Marrow Macrophages by rM180 Downregulated Allogenic CD4^+^ T Cell Activation

**Figure 3:**
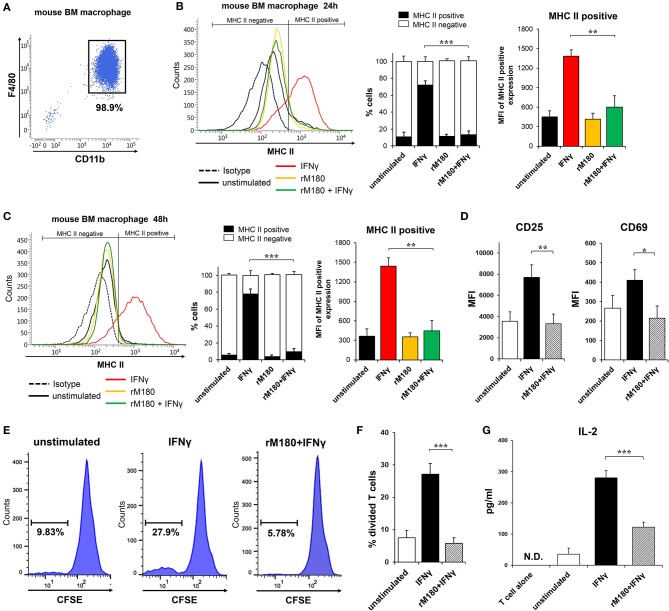
Activation of allogeneic CD4^+^ T lymphocytes by rM180-pretreated bone marrow macrophages. **(A)** Purity of bone marrow macrophages isolated from C57BL/6 mice. Bone marrow macrophages were incubated with rM180 for 24 h, washed, and stimulated with IFNγ for 24 h **(B)**, and 48 h **(C)**. **(B,C)** MHC II surface expression of mouse bone marrow macrophages was evaluated by flow cytometry. Representative histograms are shown in left panel. Quantification of cells expressing MHC II (MHC II positive cells) or not (MHC II negative cells) is shown in median panel. Quantification of histograms is shown in right panel. Bar graphs represent the mean fluorescence intensity (MFI) of MHC II positive expression. **(D–G)** Activation of allogeneic CD4^+^ T cells (BALB/c mice, H2-D^d^) in a mixed leukocyte reaction (MLR; bone marrow macrophage to T cell ratio of 1:1) with or without rM180-incubated bone marrow macrophages (C57BL/6 mice, H2-D^b^). **(D)** CD25 and CD69 mean fluorescence intensity (MFI) in CD4^+^ T cells was analyzed by flow cytometry. **(E,F)** CD4^+^ T cells were labeled CFSE, and then co-cultured with bone marrow macrophages. After 48 h, the proliferation of CD4 positive-gated cells was assessed by flow cytometry analysis of CFSE dilution. Histograms show representative data **(E)**. Bar graphs represent the percentage of divided T cells **(F)**. **(G)** IL-2 production by CD4^+^ T cells cocultured with rM180-incubated bone marrow macrophages was analyzed in culture supernatants at 72 h. N.D., not detected. The significance of differences between groups was determined by one-way ANOVA/Tukey's test: ^*^P < 0.05; ^**^P < 0.01; ^***^P < 0.001 vs. IFNγ. Data represent means ± SD. Similar results were obtained in three independent experiments.

MHC II presentation by macrophages is central for priming the adaptive immune response in CD4^+^ T lymphocytes. We therefore explored the capacity of rM180 amelogenin-stimulated bone marrow macrophages from C57BL/6 mice to activate allogeneic splenic CD4^+^ T cells isolated from BALB/c mice. Bone marrow macrophages were isolated with purity more than 90% (Figure 3A). Similar to the results depicted in Figure 1, the MHC II cell surface expression of mouse bone marrow macrophages from C57BL/6 mice was also inhibited in cells stimulated with IFNγ pretreated with rM180 compared to cells stimulated only with IFNγ for 24 or 48 h (Figures 3B,C). Next, the expression of T cell activation markers CD25 and CD69, carboxyfluorescein succinimidyl ester (CFSE)-based T cell proliferation assay, and IL-2 production by CD4^+^ T lymphocytes were measured by mixed lymphocyte reaction (MLR) assay to assess the induction of T cell activation. The expression of CD25 and CD69 (Figure 3D), and T cell proliferation ability (Figures 3E,F), as well as the production of IL-2 (Figure 3G), was significantly lower in the group stimulated with IFNγ after rM180 treatment compared with T cells that responded to macrophages stimulated only with IFNγ. These results clearly indicated that reduced MHC II expression on macrophages pretreated with rM180 impaired their capacity to activate allogenic CD4^+^ T cell responses.

### rM180 Downregulated CIITA Protein Expression in Macrophages

**Figure 4:**
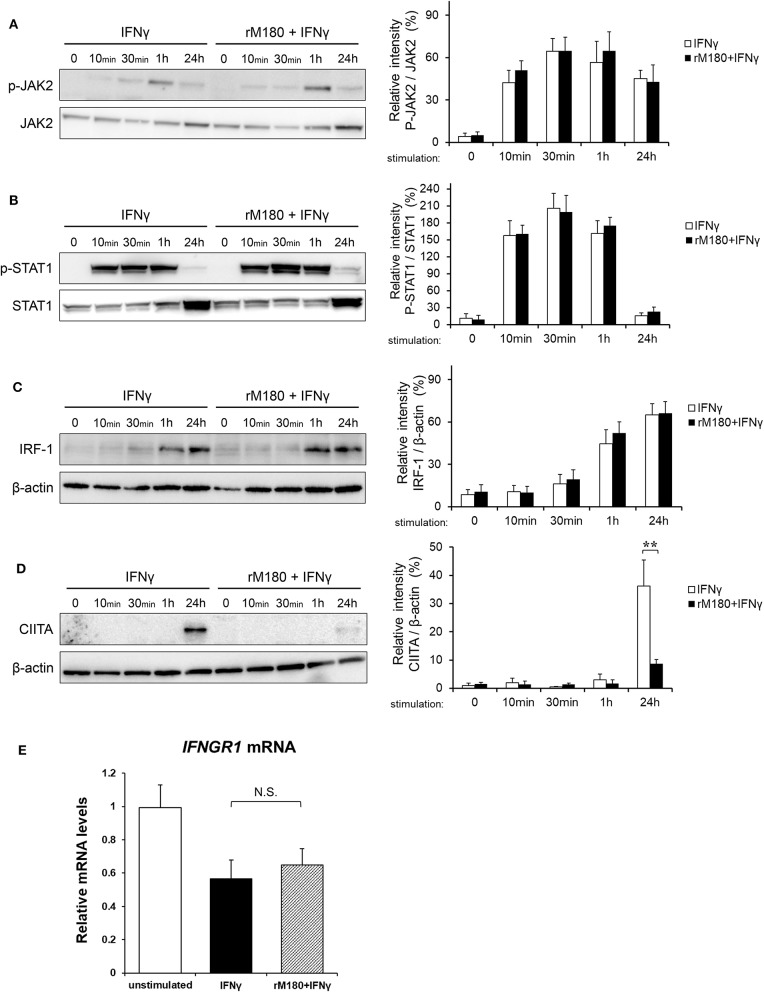
rM180 downregulates the IFNγ-induced expression of CIITA molecules in THP-1 cells. THP-1 cells were incubated with rM180 for 24 h, washed, and serum-starved for 24 h. After stimulation by IFNγ for the indicated times, whole cell lysates prepared from THP-1 cells were immunoblotted with various antibodies. β-actin was used as a control. Protein expression was quantified using the ImageJ program. Representative western blots of phosphorylated **(A)** JAK2 and **(B)** STAT1, and total **(C)** IFN regulatory factor-1 (IRF-1) and **(D)** class II transactivator (CIITA). Quantification of phosphorylated protein levels relative to total protein and total IRF-1 and CIITA protein levels relative to β-actin was carried out using ImageJ. The significance of differences between groups was determined by a two-tailed unpaired Student's test; ^**^P < 0.01. **(E)** Total RNA was isolated and analyzed for IFNγ receptor 1 (IFNGR1) mRNA expression by quantitative real-time PCR. Data are represented as fold increase in expression relative to GAPDH. The significance of differences between groups was determined by one-way ANOVA/Tukey's test. Data represent mean ± SD. Similar results were obtained in three independent experiments.

As demonstrated before, we found that rM180 amelogenin migrated rapidly into the nucleus to suppress the cell surface expression of MHC II and allogeneic T cell activation. IFNγ induction of MHC II gene expression is known to involve a process that starts with IFNγ receptor binding, JAK/STAT phosphorylation, IFN regulatory factor-1 (IRF-1) activation, and STAT/IRF-1 binding to the promoter IV (p-IV) region of CIITA. STAT/IRF-1 is a transcription activator for CIITA that initiates MHC II transcription, which in turn activates MHC II translation. To identify the signaling points that rM180 influences in this pathway, western blotting was performed. rM180 did not affect the phosphorylation of JAK or STAT1 downstream of IFNγ receptor signaling or IRF-1 activation (Figures 4A–C). However, rM180 markedly suppressed CIITA protein expression at 24 h after stimulation with IFNγ (Figure 4D). In addition, quantitative real-time PCR demonstrated that rM180 did not affect IFNγ receptor gene expression on macrophages (Figure 4E). These results clearly indicated that rM180 amelogenin inhibits CIITA protein expression and consequently downregulates MHC II expression, as shown in Figure 1.

### rM180 Appeared to Inhibit CIITA Transcription by Suppressing Euchromatin in Macrophages

**Figure 5:**
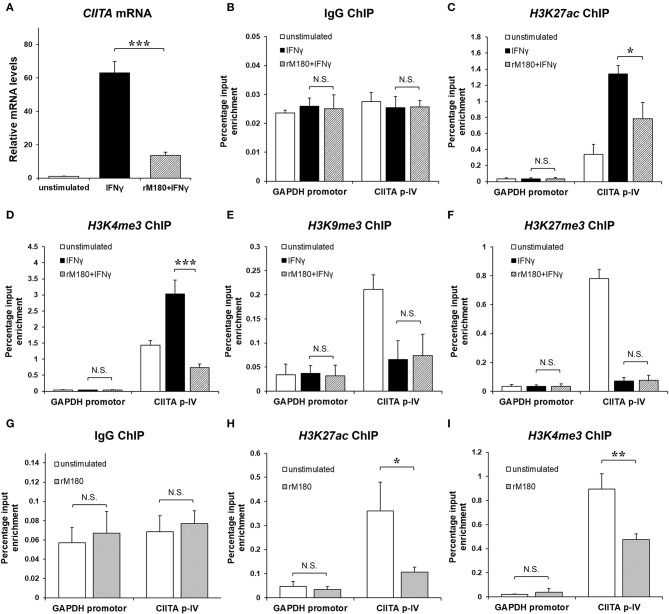
rM180 suppresses H3K27 acetylation and H3K4 trimethylation at the promoter IV region of CIITA prior to IFNγ stimulation in THP-1 cells. THP-1 cells were incubated with rM180 for 24 h, washed, and stimulated with IFNγ for 24 h. **(A)** Total RNA was isolated and analyzed for CIITA mRNA expression by quantitative real-time PCR. Data are presented as fold increase in expression relative to GAPDH. **(B–F)** ChIP assays were performed to check the IgG negative control **(B)** H3K27ac **(C)**, H3K4me3 **(D)**, and H3K9me3 **(E)**, and H3K27me3 **(F)** enrichment at the CIITA promoter IV (p-IV). **(G–I)** ChIP assays were performed to check the IgG negative control **(G)**, H3K27ac **(H)**, H3K4me3 **(I)** enrichment at the CIITA p-IV after treating with or without rM180 for 24 h. Quantification of the data was carried out by quantitative real-time PCR using specific ChIP primers. The GAPDH promotor was taken as an additional negative control for ChIP quantitative real-time PCR. The significance of differences between groups was determined by one-way ANOVA/Tukey's test: ^*^P < 0.05; ^**^P < 0.01; ^***^P < 0.001 vs. IFNγ. Data represent mean ± SD. Similar results were obtained in three independent experiments.

rM180 amelogenin suppressed CIITA gene expression (Figure 5A). Therefore, we hypothesized that rM180 affects transcriptional regulation at the CIITA p-IV region and focuses on histone modification. Histone modification involves alterations to chromatin structure as well as transcription and gene regulation (33). DNA wraps around four core histones to form a nucleosome within the chromatin structure. A low degree of condensation in chromatin is called euchromatin, where histones undergo active acetylation and methylation, leading to loose DNA winding, RNA polymerase binding, and transcription initiation. Conversely, the most condensed DNA is referred to as heterochromatin, where a characteristic methyl modification tightly packs nucleosomes together, immobilizing DNA and inhibiting transcription. We therefore focused on the acetylation of histone H3 lysine 27 (H3K27ac), which is important for conversion to euchromatin, as well as the trimethylation of histone H3 lysine 4 (H3K4me3) (34). In addition, we examined the trimethylation of H3 lysine 9 (H3K9me3) and H3 lysine 27 (H3K27me3), which is important for conversion to heterochromatin (35, 36). The chromatin immunoprecipitation (ChIP) assay showed that IFNγ stimulation increased H3K27ac and H3K4me3 levels in the CIITA p-IV region, but both were suppressed in the group stimulated with IFNγ after rM180 treatment (Figures 5B–D). On the other hand, H3K9me3 and H3K27me3 remained unaffected (Figures 5E,F). Moreover, to determine whether rM180 modulates the epigenome prior to IFNγ stimulation, the ChIP experiments on macrophages that have only been exposed to rM180 for 24 h were performed. rM180 significantly suppressed H3K27ac and H3K4me3 levels in the CIITA p-IV region compared to the unstimulated group (Figures 5G–I). These results demonstrated that chromatin structural remodeling by rM180 amelogenin inhibits the formation of euchromatin and subsequent CIITA transcription prior to IFNγ stimulation.

## Discussion

**Figure 6:**
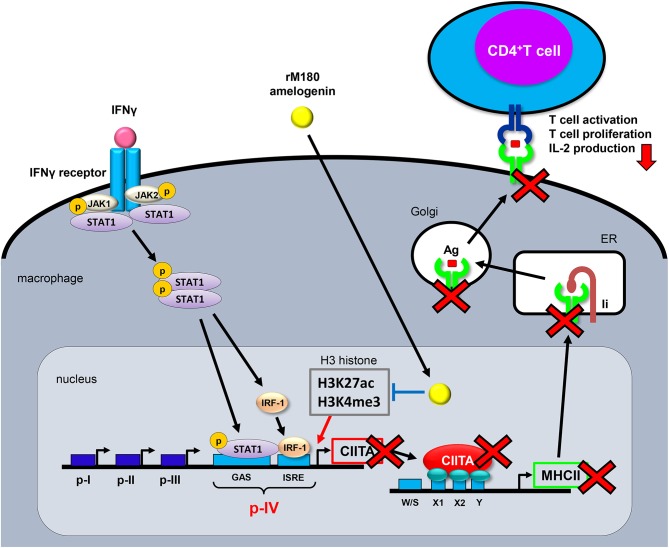
Proposed model for the MHC II inhibition mechanism by rM180 amelogenin in macrophages. Upon IFNγ binding to its receptor and inducing the JAK/STAT signaling pathways, the JAK/STAT is phosphorylated, IRF-1 is activated, and STAT/IRF-1 binds to the p-IV region of CIITA. STAT/IRF-1 is a transcription activator for CIITA that initiates MHC II transcription. rM180 amelogenin migrates early into macrophage nuclei to inhibit IFNγ-induced CIITA transcription through the suppression of H3K27ac and H3K4me3 on histone H3 within the CIITA p-IV region, thereby decreasing MHC II gene transcription. This causes a reduction in the cell surface expression of MHC II molecules, which results in the attenuation of CD4^+^ T cell activity. Ii, invariant chain; ER, endoplasmic reticulum; Ag, antigen; IRF-1, IFN regulatory factor-1; CIITA, class II transactivator; GAS, IFN-γ activated sequence; IRSE, IFN-stimulated response elements.

In the present study, we reported that rM180 amelogenin migrates rapidly into macrophage nuclei to selectively inhibit MHC II cell surface expression and attenuate T cell activation by suppressing histone H3 acetylation of lysine 27 and trimethylation of lysine 4, both of which are crucial for CIITA transcriptional activation in the promoter IV region of CIITA (Figure 6). This mechanism mediated by amelogenin appeared to be widely conserved across species.

In general, adequate control of inflammation is essential for the initiation of tissue regeneration, and macrophages play an important role both during the onset of inflammation, such as in periodontitis, as well as in wound healing after treatment. Macrophages can be broadly divided into two groups, and are classified as inflammation-mediating M1 cells or wound-healing M2 cells. M1 macrophages can be stimulated by IFNγ alone, or synergistically by IFNγ with other cytokines or bacterial components such as lipopolysaccharide (LPS). Activated M1 macrophages enhance MHC II expression and produce inflammatory factors such as inducible nitric oxide synthase (iNOS), tumor necrosis factor-alpha (TNF-α), IL-1β, and IL-6 (37, 38). M1 macrophages thus play a role in inducing Th1 response and supporting biological defense mechanisms directed against intracellular parasitic pathogens or tumors (39, 40). In contrast, M2 macrophages are induced by IL-4 and IL-13 and produce anti-inflammatory cytokines such as IL-10, transforming growth factor-beta (TGF-β), and VEGF. Therefore, M2 macrophages play a central role in angiogenesis, scavenging, resolution of inflammation, and transition to tissue repair (41–44). As such, macrophages are involved in both the destruction and regeneration of tissues, and play an important role within the interface of inflammation and tissue regeneration. In previous studies, we reported that time-dependent expression of the M2 markers CD163 and CD206 was upregulated when macrophages were stimulated with rM180 amelogenin, and that cell morphology was changed to a spindle shape, promoting differentiation into M2 macrophages (45). Additionally, inflammatory gene expression such as TNF-α, IL-6 induced by LPS was rapidly induced at 4 h post-stimulation, and returned to baseline at 24 h in the amelogenin pretreatment group, while in macrophages stimulated with LPS alone, such gene expression prolonged up to 96 h. In the amelogenin pretreatment group, the expression of anti-inflammatory genes, on the contrary, increased at 8 h post-LPS stimulation (46). Thus, amelogenin contributes to early resolution of inflammation and promotes wound healing by temporarily enhancing the initial macrophage inflammatory response and reducing the duration of inflammation.

In the present study, we found that rM180 amelogenin underwent translocation to the nucleus within 5 min, inhibiting the transcriptional activity of CIITA via H3K27ac and H3K4me3 on histone H3 within the CIITA p-IV region (Figure 6). This is a novel finding, with no other prior studies having described amelogenin translocation to the nucleus and alterations in chromatin structure. While there have been numerous studies conducted regarding amelogenin-induced cellular function (47–49), the molecules that bind to amelogenin at the cell membrane and the signaling pathways that are activated to affect cellular function remain unclear. Therefore, various studies have been conducted to identify amelogenin's binding partners. Molecules currently known to associate with amelogenin include lysosome-associated membrane glycoprotein 1 (LAMP-1), CD63 antigens, annexin A2, sialic acid-binding Ig-like lectins (Siglec-10), cytoskeletal proteins (actin, vimentin, tubulin), nuclear proteins, etc (50–56). Following this line of inquiry, we also conducted a screen for amelogenin-binding molecules, and successfully identified glucose-regulated protein 78 (GRP78) as a binding partner (32). GRP78 is a molecular chaperone that belongs primarily to the family of vesicular heat shock proteins, but functionally has also been noted to act as a receptor. We previously found that rM180 amelogenin associates with GRP78 following rM180 treatment, and that the formation of this complex leads to early uptake into the cytoplasm and import into the nucleus (32). Furthermore, we found that when GRP78 was strongly expressed, the migration activity of periodontal ligament cells was strongly promoted by rM180 stimulation (57). A possible mechanism for this sequence of events involves the amelogenin–GRP78 complex facilitating the Rho family of GTPases, Rac1 activation to promote lamellipodia formation and providing a driving force for cell migration (57). Other studies also demonstrated that GRP78 inhibits T cell proliferation by suppressing MHC II expression on dendritic cells, and concluded that GRP78 in antigen-presenting cells is an immune regulatory molecule that assists in the resolution of inflammation (58). Taken together, it is possible that amelogenin is transported into the nucleus together with molecular chaperone GRP78, thereby inhibiting euchromatin formation in the CIITA p-IV region, leading to the suppression of MHC II expression.

While full-length amelogenin was used for the present study, amelogenin is also known to include over 14 isoforms that result from splice variants of precursor mRNA. Classic alternative splice variants such as leucine-rich amelogenin peptide (LRAP) and tyrosine-rich amelogenin peptide (TRAP) are known to induce different cellular functions, and significant attention has been given to their biological significance and roles (59, 60). Although the inhibition of antigen presentation functions by LRAP and TRAP should be investigated further, it seems certain that full-length amelogenin promotes wound healing by macrophages. In the field of general regenerative medicine, the ECM has been applied for the purpose of accelerated wound healing and regeneration, and, in particular, the EMD containing amelogenin as the main component has been used to treat refractory pressure ulcers under the trade name Xelma® (61). Thus, such clinically observed acceleration of wound healing for hard-to-heal ulceration by amelogenin may be partially mediated by the mechanism elucidated in this study. Since amelogenin exhibits tolerogenic immunosuppression, it can also be used as a preventive medication against immunological rejection during organ transplantation in future applications.

In conclusion, the present study shows that amelogenin suppresses MHC II expression by altering chromatin structure and inhibiting CIITA p-IV transcription activity, and attenuates subsequent T cell activation. Amelogenin is being used clinically in periodontal tissue regeneration therapy and its effectiveness has been proved; in addition, the use of recombinant protein is safe because it is biologically derived protein. Therefore, it may also be used in future as an immunosuppressant with minimal side effects for organ transplantation or MHC II-linked autoimmune diseases such as type I diabetes, multiple sclerosis, and rheumatoid arthritis, among others.

## Data Availability Statement

All datasets generated for this study are included in the article/supplementary material.

## Ethics Statement

The animal study was reviewed and approved by Animal Care and Use Committee of Kyushu University.

## Author Contributions

The experiments were designed and conceived by TSa, UT, TF, and FN. The experiments were performed by KY, HY, RA, TSi, YN, YW, and CH. Data was analyzed by KY, TSa, UT, TF, and FN. Materials, reagents were facilitated by TT. All experiments were supervised by FN. KY, TSa, and FN wrote the manuscript. All authors read and approved the final manuscript.

## Conflict of Interest

The authors declare that the research was conducted in the absence of any commercial or financial relationships that could be construed as a potential conflict of interest.
